# A case of a readmitted patient who recovered from COVID-19 in Chengdu, China

**DOI:** 10.1186/s13054-020-02877-8

**Published:** 2020-04-16

**Authors:** Xiao-jin Li, Zhong-wei Zhang, Zhi-yong Zong

**Affiliations:** 1 Intensive Care Unit, West China Hospital, Sichuan University, 610041, Chengdu, China; 2 Center for Infectious Diseases, West China Hospital, Sichuan University, 610041, Chengdu, China

Dear Editor,

A large number of coronavirus disease 2019 (COVID-19) studies have emerged recently, and most of them investigated the clinical features, epidemiological treatment, and clinical outcomes of these patients [1–4]. There is no confirmed evidence about patients (non-medical personnel) readmitted after discharge.

A 41-year-old male who had been to Wuhan City on January 8, 2020, returned to Chengdu on January 22 and was confirmed to have COVID-19 on January 26 at the Fifth People’s Hospital of Chengdu City. He was immediately transferred to the ICU of Chengdu’s Infectious Disease Center. A physical examination revealed that his body temperature was 38 °C, his pulse was 118 beats per minute, his blood pressure was 121/88 mmHg, and his pulse oxygen saturation was 90% (oxygen treatment, FIO2, 50%). The patient was critically ill. After admission, atomization inhalation to human recombinant interferon antiviral therapy, traditional Chinese medicine, and oxygen therapy were administered. On February 3, the criteria had to be met for hospital discharge; the image of chest computed tomographic (CT) scan improved from the first scan, as shown in Fig. 1. He was discharged and went back home for a 2-week quarantine. On February 21, because of reoccurrence of chest pain and cough, he went to our hospital; his RT-PCR tests were performed on nasal swabs, sputum, and stool [1]; and all detection results were positive; however, RT-PCR throat swabs were negative, B cells increased, and NK cells decreased. Chest CT images were obtained on February 22, which revealed that there were scattered patches and ground-glass opacity on both lungs, as shown in Fig. 2 with a red arrow. The typical imaging feature of the chest CT scan in the patient was the white “Septal Line” marked by the yellow arrow in Figs. 1 and 2, suggesting that cellulosic exudation occurred on the surface of the lung lobes. In the dynamic imaging, these white lines are visible in Fig. 2, and this provides evidence for us to judge the reoccurrence of COVID-19. The patient was readmitted to the isolated ward until February 29. The symptoms improved, and SARS-CoV-19 RT-PCR tests of stool samples were positive. The patient is still in our hospital for treatment, and his condition is now stable.

**Fig. 1:**
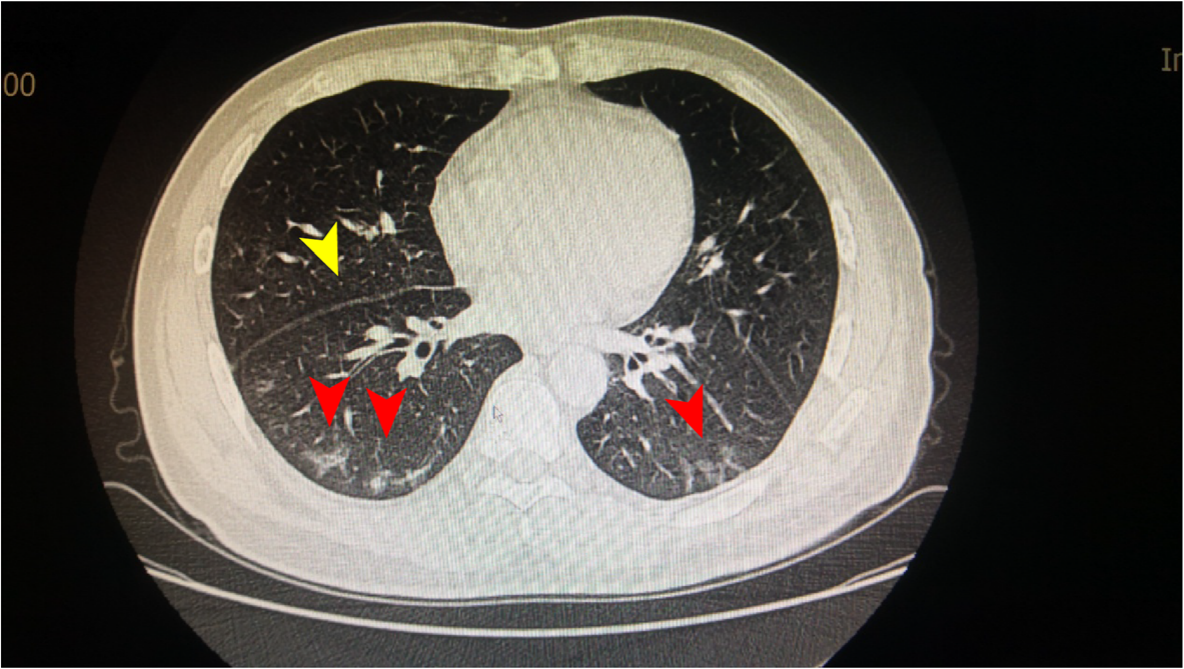
Image taken on February 3, 2020. Flaky, ground-glass opacity close to the visceral pleura is marked by the red arrows. The white “Septal Line” is marked by a yellow arrow

**Fig. 2:**
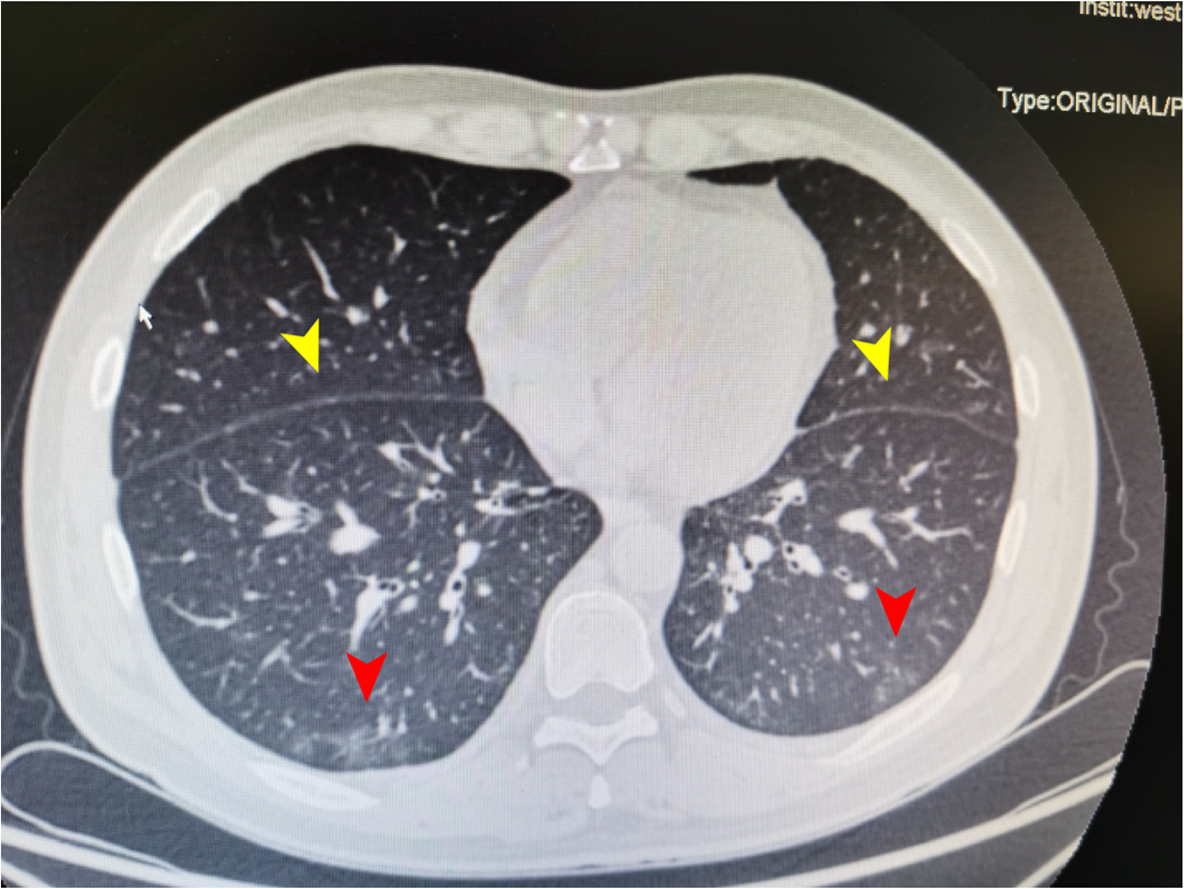
Image taken on February 22, 2020. Flaky, ground-glass opacity close to the visceral pleura is marked by the red arrows. The white “Septal Line” is marked by the yellow arrows

The novel coronavirus (2019-nCoV) pneumonia is highly infectious, insidious, and repeated [2–4]. In our report, after 2–3 weeks of treatment, the patient was discharged. On February 22, the patient’s symptoms reoccurred, CT and nucleic acid tests for 2019-nCoV suggested that the virus in the body may not have been completely cleared from the patient on February 3 when he was discharged, and current criteria for hospital discharge (7th edition) may need to be re-evaluated and further adjusted. It has been reported that a follow-up found that RT-PCR tests of 3 medical staff members were positive 5 to 13 days after a rehabilitation home quarantine [5], but there was no confirmed evidence about patients (non-medical personnel) with recurrence after discharge. We focused on this case of a “clinical cured” patient, in which the symptoms reoccur and RT-PCR test for 2019-nCoV was positive; from onset to hospital readmission, the treatment course had been more than 35 days. Our findings suggested that some patients may be a long repeatable process.

The National Discharge Standard of COVID-19 needs to be updated, and further clinical evidence, discharge, and follow-up of COVID-19 need to be improved.

## Data Availability

The data are available from the corresponding author.

## References

[1] Wang D, Hu B, Hu C, Zhu F, Liu X, Zhang J (2020). Clinical characteristics of 138 hospitalized patients with 2019 novel coronavirus-infected pneumonia in Wuhan, China. JAMA.

[2] Liew MF, Siow WT, MacLaren G, See KC (2020). Preparing for COVID-19: early experience from an intensive care unit in Singapore. Crit Care.

[3] Zhu N, Zhang D, Wang W, Li X, Yang B, Song J (2020). A novel coronavirus from patients with pneumonia in China, 2019. N Engl J Med.

[4] Huang C, Wang Y, Li X, Ren L, Zhao J, Hu Y (2020). Clinical features of patients infected with 2019 novel coronavirus in Wuhan, China. Lancet.

[5] Lan L, Xu D, Ye G, Xia C, Wang S, Li Y, et al. Positive RT-PCR test results in patients recovered from COVID-19. JAMA. 2020. 10.1001/jama.2020.2783. 10.1001/jama.2020.2783 PMC7047852 32105304

